# Osthole Attenuates Macrophage Activation in Experimental Asthma by Inhibitingthe NF-ĸB/MIF Signaling Pathway

**DOI:** 10.3389/fphar.2021.572463

**Published:** 2021-03-22

**Authors:** Ruyi Li, Peng Song, Guofang Tang, Jianghong Wei, Lizong Rao, Libing Ma, Ming Jiang, Jianwei Huang, Qing Xu, Jingjie Wu, Qian Lv, Dong Yao, Bo Xiao, Haiming Huang, Liping Lei, Juntao Feng, Biwen Mo

**Affiliations:** ^1^ Key Laboratory of National Clinical Research Center for Respiratory Disease, Department of Respiratory and Critical Care Medicine, Xiangya Hospital, Central South University, Changsha, China; ^2^ Key Laboratory of Prevention and Treatment for Chronic Diseases by Traditional Chinese Medicine, Affiliated Hospital of Gansu University of Chinese Medicine, Lanzhou, China; ^3^ Innovation Research Institute of Traditional Chinese Medicine, Shanghai University of Traditional Chinese Medicine, Shanghai, China; ^4^ Department of Respiratory and Critical Care Medicine, Affiliated Hospital of Guilin Medical University, Guilin, China; ^5^ Laboratory of Pulmonary Diseases, Guilin Medical University, Guilin, China

**Keywords:** osthole, macrophages, asthma, NF-ĸB, MIF 46

## Abstract

Inhibition of activated macrophages is an alternative therapeutic strategy for asthma. We investigated whether a coumarin compound, osthole, isolated from *Cnidium* *monnieri* (L.) Cuss, alleviated macrophage activation *in vivo* and *in vitro*. Osthole could reduce expression of a marker of activated macrophages, cluster of differentiation (CD)206, in an ovalbumin-challenge model of asthma in mice. Osthole could also inhibit infiltration of inflammatory cells, collagen deposition and production of proinflammatory cytokines [interleukin (IL)-1β, tumor necrosis factor-ɑ, macrophage migration inhibitory factor (MIF)] in asthmatic mice. *In vitro*, expression of phosphorylated-IĸBɑ, MIF and M2 cytokines (Ym-1, Fizz-1, arginase-1) in IL-4-induced macrophages decreased upon exposure to the NF-ĸB inhibitor MG-132. In our short hairpin (sh)RNA-MIF-knockdown model, reduced expression of M2 cytokines was detected in the IL-4 + shRNA-MIF group. Osthole could attenuate the proliferation and migration of an IL-4-induced rat alveolar macrophages line (NR8383). Osthole could reduce IL-4-induced translocation of nuclear factor-kappa B (NF-ĸB) in NR8383 cells. Collectively, our results suggest that osthole ameliorates macrophage activation in asthma by suppressing the NF-ĸB/MIF signaling pathway, and might be a potential agent for treating asthma.

## Introduction

Asthma is a complex, heterogeneous pulmonary disease that affects ∼300 million people worldwide (Peters et al., 2006; Siracusa et al., 2015). The key features of asthma are infiltration of inflammatory cells, reversible airflow obstruction, mucus secretion and bronchospasm (Medoff et al., 2008). The economic costs of asthma management are high and targeting asthma symptoms in the clinic are limited (Levy et al., 2015). Hence, development of novel, efficacious medications for asthma treatment is important.

Macrophages participate in immune homeostasis in the lung through phagocytosis and chemokine production in response to antigens (Moreira and Hogaboam, 2011). Macrophages (especially M2 macrophages) were discovered originally as a response to the T-helper type 2 (Th2) cytokine interleukin (IL)-4 (Stein et al., 1992). M2 macrophages can be derived from resting macrophages by exposure to IL-4 or IL-13. M2 macrophages are characterized by expression of arginase (Arg)-1, Ym-1, Fizz-1, mannose receptors and scavenger receptors (Gordon, 2003). One of the most prevalent animal asthmatic model used is OVA-sensitized and challenged C57BL/6 mice model, which can effectively induce asthma features, such as airway inflammation, airway smooth muscle mass and goblet cell hyperplasia. These properties are similar to human asthma charicteristics (Kim et al., 2019). Recent studies have shown that depletion of pulmonary alternatively activated macrophages can attenuate airway inflammation in people suffering from asthma (Lee et al., 2015). Therefore, inhibition of activation of Th2-related macrophages may provide an alternative strategy for the clinical treatment of asthma.

*Cnidii monnieri* Fructus are the dried fruits of *Cnidium monnieri* (L.) Cusson. *C. monnieri* Fructus is an important traditional Chinese medicine formulation and used to treat osteoporosis, sexual dysfunction, asthma and skin ailments (Baba et al., 1992). Osthole (also known as “osthol”) is found in various plants, including *Cnidium monnieri*. The pharmacologic activities of osthole include anti-oxidative (Zhang et al., 2011), anti-inflammatory (Liu et al., 2005), anti-allergic (Chiu et al., 2008), and anti-diabetes mellitus (Liang et al., 2009) effects. Recent studies have shown that osthole can attenuate the ovalbumin (OVA)-induced inflammation observed in allergic asthma by inhibiting nuclear factor-kappa B (NF-ĸB) activation (Wang et al., 2017). Data on the role of osthole in macrophage activation in asthma patients are lacking.

Here, we demonstrated that osthole can reduce macrophage activation in OVA-challenged mice and an IL-4-induced macrophage cell line by targeting the NF-ĸB/macrophage migration inhibitory factor (MIF) pathway.

## Materials and Methods

### Ethical Approval of the Study Protocol

The study protocol was approved by the Animal Care and Use Committee of Shanghai University of Traditional Chinese Medicine (PZSHUTCM18120712, Shanghai, China).

### Chemicals and Reagents

**FIGURE 1:**
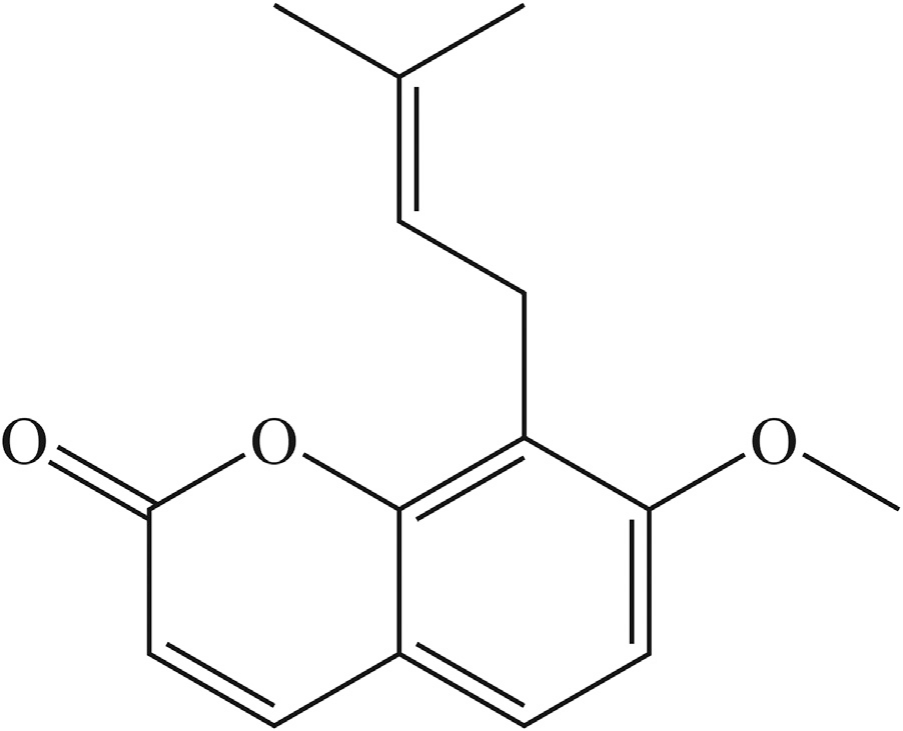
Osthole alleviates OVA-induced macrophage activation and MIF production. **(A)** Staining (H&E, Masson, AB-PAS) was undertaken to evaluate infiltration of inflammatory cells, collagen deposition and GC metaplasia (×200; scale bar, 100 μm) in lung tissues. **(B)** The total number of infiltrated cells, lymphocytes and eosinophils in BALF was increased in OVA groups compared with that in the control group. Treatment with osthole (15, 40 mg/kg) or DEX (1 mg/kg) decreased the numbers of total cells, lymphocytes and eosinophils significantly. **(C)** Osthole and DEX reduced OVA-induced increased expression of TNF-α, IL-1β and MIF in BALF. **(D)** mRNA expression of MIF was decreased in asthmatic mice after treatment with osthole or DEX. **(E,G)** Immunofluorescence for CD206 was conducted to assess accumulation of activated macrophages in lung tissues (×400; scale bar, 50 μm). Decreased expression of CD206 was noted in OVA + osthole and OVA + DEX groups compared with that in the OVA group. **(F,H)** Osthole and DEX decreased OVA-induced increased protein expression of MIF in lung tissues as assessed by immunofluorescence (×400; scale bar, 50 μm). Data are the mean ± SD, n = 5 mice each group, *** *p* < 0.001 *vs*. control; ^*p* < 0.05 *vs*. OVA group; ^^*p* < 0.01 *vs*. OVA group; ^^^*p* < 0.001 *vs*. OVA group; & *p* < 0.05 *vs*. OVA + Osthole group (15 mg/kg); *p* < 0.01 *vs*. OVA + Osthole group (15 mg/kg).

Osthole (Figure 1) was purchased from Shanghai Standard Technology (Shanghai, China). OVA (grade V) was obtained from Sigma–Aldrich (Saint Louis, MO, United States). Dexamethasone (Dex) was purchased from Changle Pharmaceuticals (Henan, China). Recombinant murine IL-4 was obtained from Peprotech (Rocky Hill, NJ, United States). MG-132 (NF-ĸB inhibitor) was purchased from MedChemExpress (Monmouth Junction, NJ, United States). ELISA kits for IL-1β, tumor necrosis factor (TNF)-ɑ and MIF for mice were obtained from R&D Systems (Minneapolis, MN, United States). Western-blot and primary antibodies for immunofluorescence staining were purchased from Cell Signaling Technology (Danvers, MA, United States). All other chemicals were of reagent grade.

### Animals

Specific pathogen-free female C57BL/6 mice (6–8 weeks) were purchased from the Experimental Animals Center of Shanghai University of Traditional Chinese Medicine. Before experimentation, all mice were maintained under standard conditions, and survived on distilled water and standard chow *ad libitum*, for 7 days.

### Sensitization and Challenge With OVA

We wished to build an asthma model using OVA in mice. Briefly, mice were sensitized by injection (i.p.) with OVA (100 µg) and aluminum hydroxide (2 mg) in 200 µl of physiologic (0.9%) saline on days 0, 7, 14 and followed by administration (i.n.) of OVA (20 mg/ml) in 50 µl of 0.9% saline from day-15 to day-28. Non-OVA-challenged mice were sensitized and challenged with 0.9% saline alone. Mice were divided into five groups of six: control, OVA, OVA + osthole (15, 40 mg/kg), and OVA + Dex (1 mg/kg). Osthole (15, 40 mg/kg) was given (i.p.) 1 h before OVA challenge from day-22 to day-28 for 7 days. Dex (1 mg/kg) was given (i.p.) 1 h before OVA challenge from day-15 to day-28. For the control group, mice were given an equal volume of 0.9% saline for 14 days. Mice were sacrificed to determine the pathophysiological and immunological features of asthma 24 h after the final challenge.

### Bronchoalveolar Lavage Fluid Collection

BALF was collected by lavage of the lung twice with 0.5 ml of cold physiologic saline *via* a tracheal catheter. BALF was centrifuged immediately (2,000 g, 5 min, 4°C). The supernatant was used for cytokine measurements. The cell pellet was resuspended in 0.5 ml of phosphate-buffered saline (PBS) and used for total and differential cell counts, which were measured by H&E-stained cytocentrifuges.

### Measurement of Cytokine Levels in BALF and Cell Supernatants

Concentrations of MIF, IL-1β and TNF-α in BALF and the concentration of MIF protein in supernatants of NR8383 cells were measured by ELISAs according to manufacturer (R&D Systems) instructions.

### Real-Time Reverse Transcription-Quantitative PCR

Total RNA was isolated from lung tissues and lysates of NR8383 cells by TRIzol^®^ Reagent according to manufacturer (Invitrogen, Carlsbad, CA, United States) instructions. Reverse transcription was carried out with the PrimeScript^®^ RT Reagent Kit (TaKaRa Biotechnology, Shiga, Japan). Next, RT-qPCR was done with the SYBR^®^ Green Premix Ex Taq kit (TaKaRa Biotechnology) on a LightCycler^®^ 480 system (Roche, Basel, Switzerland). mRNA expression of target genes was normalized to β-actin expression in the same sample. The primers we used (forward and reverse, respectively) were: 5′-CCA​GAA​CCG​CAA​CTA​CAG​TAA​GC-3′ and 5′-TTG​GCA​GCG​TTC​ATG​TCG​TAA​TAG-3′ for MIF (mouse); 5′-ATC​ACT​ATT​GGC​AAC​GAG​CGG​TTC-3′ and 5′-CAG​CAC​TGT​GTT​GGC​ATA​GAG​GTC-3′ for β-actin (mouse); 5′-TCC​GTG​CCA​GAG​GGG​TTT​CTC-3′ and 5′-GGG​TCG​CTC​GTG​CCA​CTA​AAA​G-3′ for MIF (rat); 5′-CTG​AGA​GGG​AAA​TCG​TGC​GTG​AC-3′ and 5′-AGG​AAG​AGG​ATG​CGG​CAG​TGG-3′ for β-actin (rat).

### Lung Histopathology

Lung tissues were fixed with 10% neutral formalin after mice had been sacrificed. Fixed sections were embedded in paraffin, sectioned and stained with H&E to examine infiltration of inflammatory cells. Staining [Alcian blue and periodic acid-Schiff (AB-PAS), Masson] was used to evaluate mucin-positive goblet cells (GCs) and collagen deposition.

### Culture and Transfection of Cells

The rat alveolar macrophage cell line NR8383 was purchased from Shanghai Zhong Qiao Xin Zhou Biotechnology (Shanghai, China). The peritoneal macrophage cell line RAW264.7 was obtained from Shanghai Institutes for Biological Sciences (Shanghai, China).

Cells were cultured in Dulbecco’s modified Eagle’s medium (Hyclone, Julich, Germany) supplemented with 10% fetal bovine serum (Gibco, Grand Island, NY, United States). All cells were grown at 37°C in humidified air in an atmosphere of 5% CO_2_. Cells were treated with the indicated concentration of osthole according to experimental requirements; the same volume of DMEM was used as the solvent control. A short hairpin RNA (shRNA) sequence targeting *MIF* in mice was inserted into the PGMLV-hU6-MCS-CMV-ZsGreen1-PGK-Puro-WPRE vector to generate the MIF-si plasmid. The sequence used to silence MIF expression was 5′-GGG​TCT​ACA​TCA​ACT​ATT​ACG-3′. The plasmid transfected with scramble siRNA (5′-TTC​TCC​GAA​CGT​GTC​ACG​T-3′) was used as a negative control. Lentiviral particles targeting MIF were transfected into RAW264.7 cells. Transfected cells were obtained from Genomeditech (Shanghai, China). The transfection efficiency was confirmed by real-time RT-qPCR (Supplementary Figure S1).

### Cell-Viability Assay

Cell viability was measured using the MTT Cell Viability Assay Kit (Beyotime Biotechnology, Beijing, China) on 96-well plates. After drug treatment, cells were incubated with 20 μl of MTT solution (5 mg/ml) for 4 h at 37°C, followed by replacement of the medium with 150 µl of dimethyl sulfoxide. Absorbance was read at 570 nm on an automated microplate reader (BioTek, Winooski, VT, United States).

### Colony-formation Assay

NR8383 cells were inoculated into 12-well plates at 400 cells/well. Cells were divided into four groups (control, IL-4, IL-4 + osthole, IL-4 + Dex) and cultured in an atmosphere of 5% CO_2_ at 37°C for 14 days. Cells were stained with 0.1% crystal violet (Beyotime). A digital camera (Nikon, Tokyo, Japan) was used for imaging.

### Migration Assay

Cells were placed into the upper chamber of Transwell™ inserts (Corning, Corning, NY, United States) with culture medium to detect cell migration. Culture medium was also added to the lower chamber. After 48 h of incubation at 37°C, cells migrated through the membrane were fixed with 4% formaldehyde for 30 min and stained with 0.1% crystal violet. Stained cells were imaged and counted under a microscope (Nikon).

### Western Blotting

Cells were treated with NP40 lysis buffer (Beyotime) on ice for 30 min. Lysates were centrifuged at 12,000 × *g* for 10 min at 4°C. The supernatant was collected and protein concentrations were measured with the Protein BCA Assay Kit (Beyotime). Protein samples (30 μg of total protein each) were boiled at 100°C for 5 min and separated by sodium dodecyl sulfate–polyacrylamide gel electrophoresis and transferred onto polyvinylidene difluoride (PVDF) membranes (Bio-Rad Laboratories, Hercules, CA, United States). The primary antibodies we used were anti-NF-ĸB-p65 (1:1,000 dilution), anti-MIF (1:1,000), anti-lamin-A (1:1,000), anti-IĸBα (1:1,000), anti-phosphorylated (p)-IĸBα (1:1,000), anti-Ym-1 (1:1,000), anti-Fizz-1 (1:1,000), anti-Arg-1 (1:1,000) and anti-β-actin (1:1,000). This action was followed by incubation with fluorescent-labeled secondary antibodies (1:5,000; Cell Signaling Technology). Band detection was performed on a platform from Azure Biosystems (Dublin, CA, United States). Quantification of protein levels was done by densitometry using ImageJ v5.0 (National Institutes of Health, Bethesda, MD, United States).

### Cell-Proliferation Assay

Measurement of cell proliferation was based on a colorimetric immunoassay (Abcam, Cambridge, United Kingdom) in which bromodeoxyuridine (BrdU) was incorporated into cells during DNA synthesis. All procedures were undertaken following manufacturer instructions. After preincubation, cells were labeled with BrdU for 2 h. BrdU incorporation was detected by adding an anti-BrdU antibody and the subsequent substrate reaction.

### Scratch Assay

Cells were allowed to reach a confluent monolayer in six-well plates. A conventional pipette was used to make a scratch in the cell monolayer. The monolayer was washed and incubated further. The imaging of scratches was captured by a digital camera system coupled to an inverted microscope (Nikon). Then, cells were incubated for 48 h, and the scratches were imaged by the microscope.

### Immunofluorescence

Sections were fixed using 4% paraformaldehyde for 24 h at 4°C. For immunofluorescence staining of cluster of differentiation (CD)206, MIF and NF-ĸB-p65, sections were permeabilized with 0.3% Triton X-100 in PBS for 20 min at room temperature and blocked with 5% bovine serum albumin (Beyotime) for 30 min. Then, cells were incubated with primary antibodies overnight at 4°C. The primary antibodies we used were anti-CD206 (1:350 dilution), anti-MIF (1:300) and anti-NF-ĸB-p65 (1:200). Subsequently, sections were washed with PBS and then incubated with the appropriate secondary antibody for 2 h at room temperature. The secondary antibodies we employed were anti-rabbit immunoglobulin (Ig)G (1:450 dilution) and anti-mouse IgG (1:450), both of which were from Cell Signaling Technology. 4′,6-diamidino-2-phenylindole (Invitrogen) staining was used to visualize nuclei. Finally, a laser fluorescence microscope (Nikon) was employed to view immunolabeled slides. Immunofluorescence quantification was carried out using ImageJ v5.0 (National Institutes of Health).

### Statistical Analyses

Data are the mean ± SD. Differences among groups were assessed by ANOVA and the Student’s *t*-test. Statistical analyses were undertaken using Prism 5.0 (GraphPad, San Diego, CA, United States). P < 0.05 was considered significant.

## Results

### Osthole Inhibited Macrophage Activation and MIF Production in OVA-Challenged Mice

**FIGURE 2:**
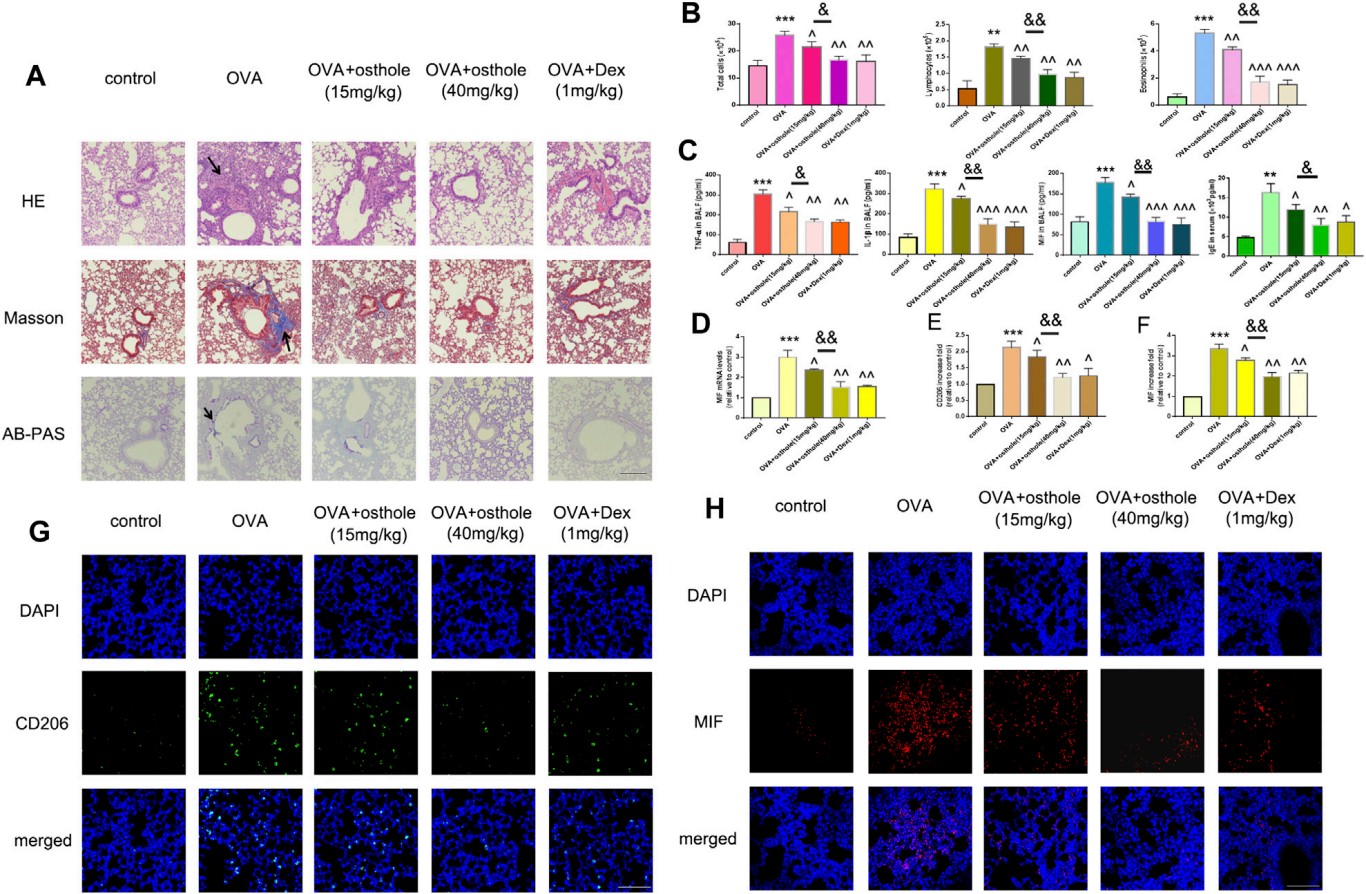
IL-4-induced macrophage activation was mediated by the NF-ĸB/MIF signaling pathway. **(A,B)** Phosphorylated and total protein expression of IĸBɑ in the presence or absence of NF-ĸB inhibitor MG-132 was determined by western blotting. **(C)** MIF secretion in supernatants was measured by ELISA. mRNA expression of MIF in macrophages was measured by RT-qPCR. **(D,F)** Protein expression of the M2-type cytokines Ym-1, Fizz-1 and Arg-1 was determined by western blotting. **(E,G)** Protein expression of Ym-1, Fizz-1 and Arg-1 in MIF-knockdown RAW264,7 cells was measured by western blotting. Data are the mean ± SD. *p < 0.05 *vs*. control; **p < 0.01 *vs*. control; ***p < 0.001 *vs*. control; ^*p* < 0.05 *vs*. IL-4 group; ^^*p* < 0.01 *vs*. IL-4 group.

We wished to determine the role of osthole in an experimental model of asthma. We detected airway remodeling and airway inflammation in an OVA-challenged mice after osthole administration. First, staining (H&E, Masson, AB-PAS) showed that inflammation infiltration, collagen deposition and GC metaplasia decreased strongly in OVA-challenged asthmatic mice after treatment with osthole (15 and 40 mg/kg) and Dex (1 mg/kg) compared with that upon administration of 0.9% saline (Figure 2A). Second, we counted the number of inflammatory cells (Figure 2B) and protein expression of proinflammatory cytokines in BALF and IgE level in serum (Figure 2C) by ELISA: we documented a decrease in osthole and Dex groups compared with that in the OVA group. With regard to macrophage activation, we counted the number of CD206^+^ cells in lung tissues by fluorescence. The number of CD206^+^ cells was reduced in osthole and Dex groups (Figures 2E,G). Moreover, MIF expression in lung tissues and BALF decreased in asthmatic mice after treatment with osthole or Dex (Figures 2D,F,H). In osthole groups, the decrease in inflammation, macrophage activation and MIF production was in a dose-dependent manner. Airway inflammation, the number of CD206^+^ cells and MIF expression increased in the OVA group compared with that in the control group.

### IL-4-Induced Macrophage Activation was Associated With the NF-ĸB/MIF Signaling Pathway *In Vitro*

**FIGURE 3:**
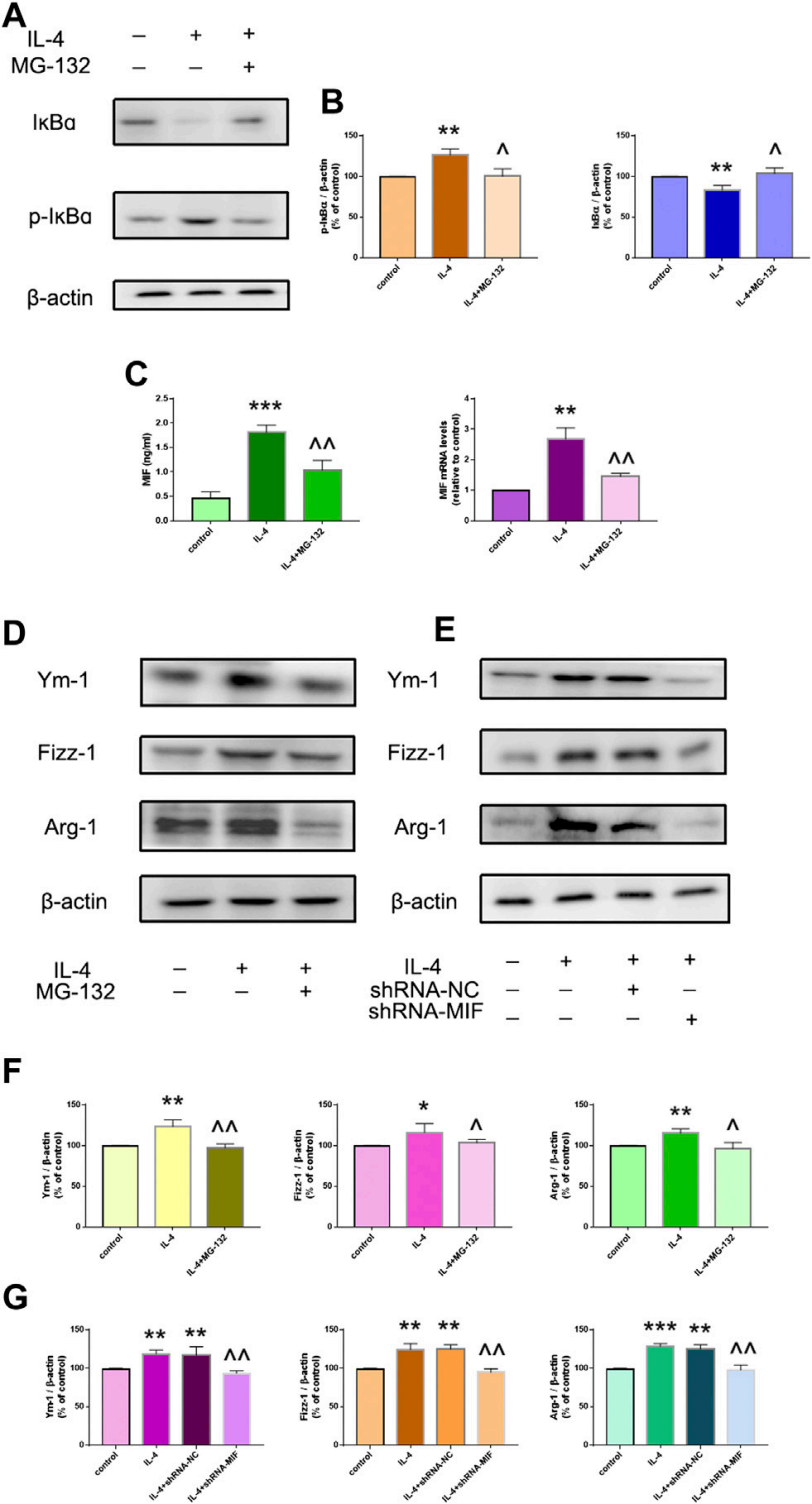
Osthole reduces IL-4-mediated macrophage activation *in vitro*. **(A)** Alveolar macrophages were treated with osthole (0, 1, 5, 10, 25, 50, 100 or 200 μM) for 48 h. The toxicity of osthole on macrophages was detected by the MTT assay. **(B**,**C)** The colony-formation assay and BrdU cell-proliferation assay were used to analyze cell proliferation. Osthole (50 μM) and DEX (100 nM) decreased IL-4-induced macrophage proliferation significantly. **(D**,**E)** The scratch assay and Transwell migration assay were used to analyze macrophage migration. Osthole and DEX reduced IL-4-induced macrophage migration. Data are the mean ± SD. ** *p* < 0.001 *vs*. control; ^^^*p* < 0.001 *vs*. IL-4 group.

We wished to assess if IL-4-induced macrophage activation was regulated by NF-ĸB/MIF signaling. NR8383 cells were pretreated with the NF-ĸB inhibitor MG-132 for 1 h before stimulation with IL-4 for 24 h. MG-132 suppressed IL-4-related phosphorylation of IĸB and MIF secretion greatly (Figures 3A–C). MG-132 could also block IL-4-induced highly expression of Ym-1, Fizz-1 and Arg-1 in NR8383 cells (Figures 3D,F). After exposure to IL-4 for 24 h, MIF-knockdown RAW264.7 cells had reduced expression of Ym-1, Fizz-1 and Arg-1 compared with that in cells transfected with scramble controls (Figures 3E,G).

### Osthole Ameliorated Macrophage Activation *In Vitro*

**FIGURE 4:**
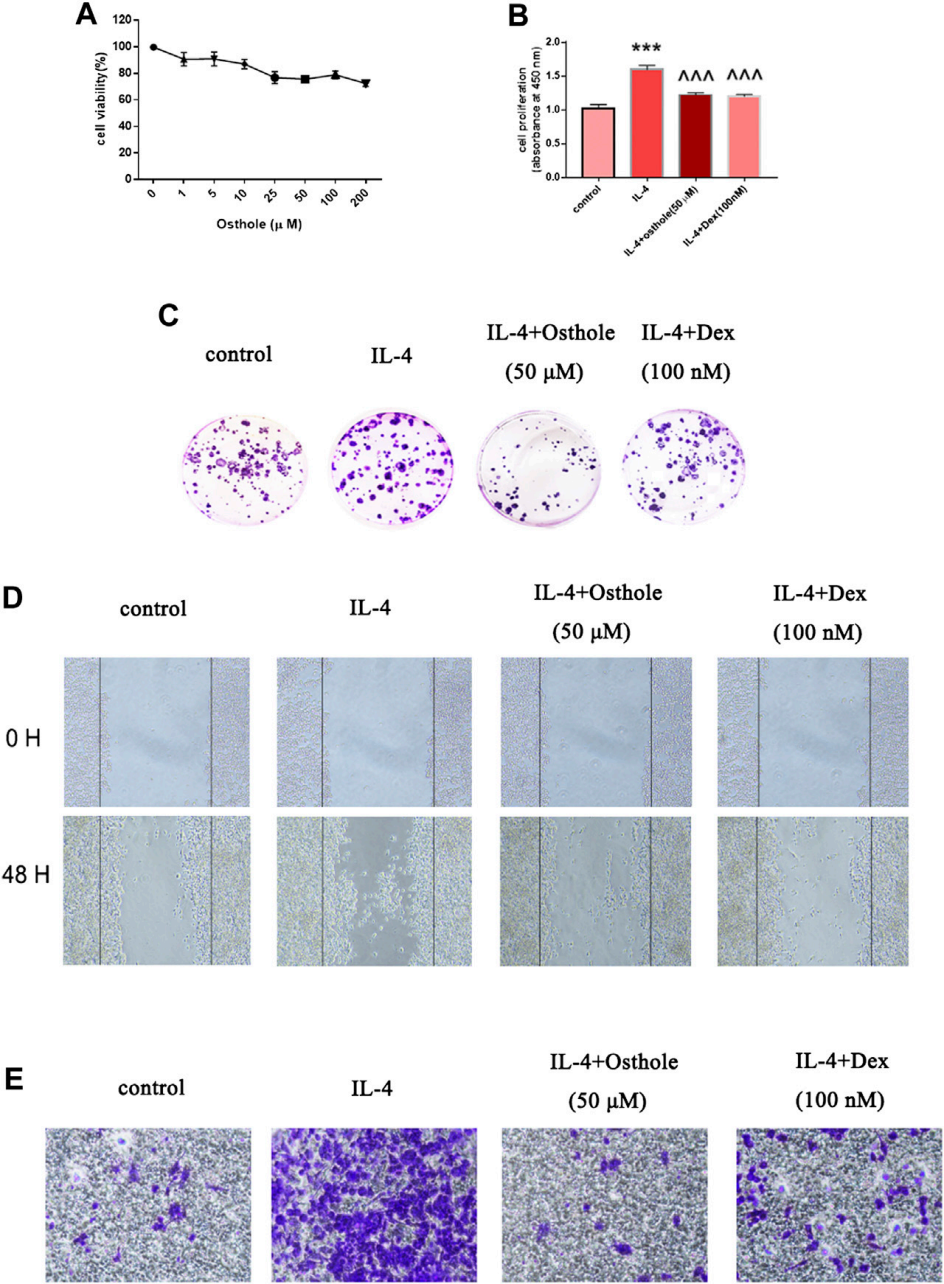
Osthole inhibits IL-4-induced NF-ĸB activation in macrophages. **(A)** Protein expression of nuclear NF-ĸB, cytoplasmic NF-ĸB and MIF was measured by western blotting. **(B)** Osthole (50 μM) could reduce IL-4-induced NF-ĸB activation and MIF production. **(C)** Translocation of the p65 subunit of NF-ĸB from the cytoplasm to nuclei was assessed by immunofluorescence (1000 × ; scale bar, 10 μm). Nuclei were stained with DAPI. Osthole decreased IL-4-induced NF-ĸB p65 translocation. Data are the mean ± SD. *p < 0.05 *vs*. control; **p < 0.01 *vs*. control; ^*p* < 0.05 *vs*. IL-4 group; ^^*p* < 0.01 *vs*. IL-4 group.

As described above, osthole inhibited accumulation of CD206^+^ cells in a mouse model of asthma. Next, we investigated if osthole inhibited macrophage activation *in vitro*. The potential toxicity of osthole to NR8383 cells was measured by the Cell Counting Kit-8 assay after incubation with osthole for 48 h (Figure 4A). Osthole did not display toxicity against NR8383 cells. The latter were preincubated with osthole (50 μM) for 1h, then stimulated with IL-4 (10 ng/ml) for 24 h. Cell proliferation was measured by the BrdU assay (Figure 4B) and colony formation (Figure 4C). Osthole treatment attenuated IL-4-induced macrophage proliferation. The scratch assay (Figure 4D) and Transwell assay (Figure 4E) showed that osthole could reduce IL-4-induced macrophage migration. These data suggested that osthole can alleviate IL-4-induced macrophage activation.

### Osthole Blocked IL-4-Induced NF-ĸB Translocation

**FIGURE 5:**
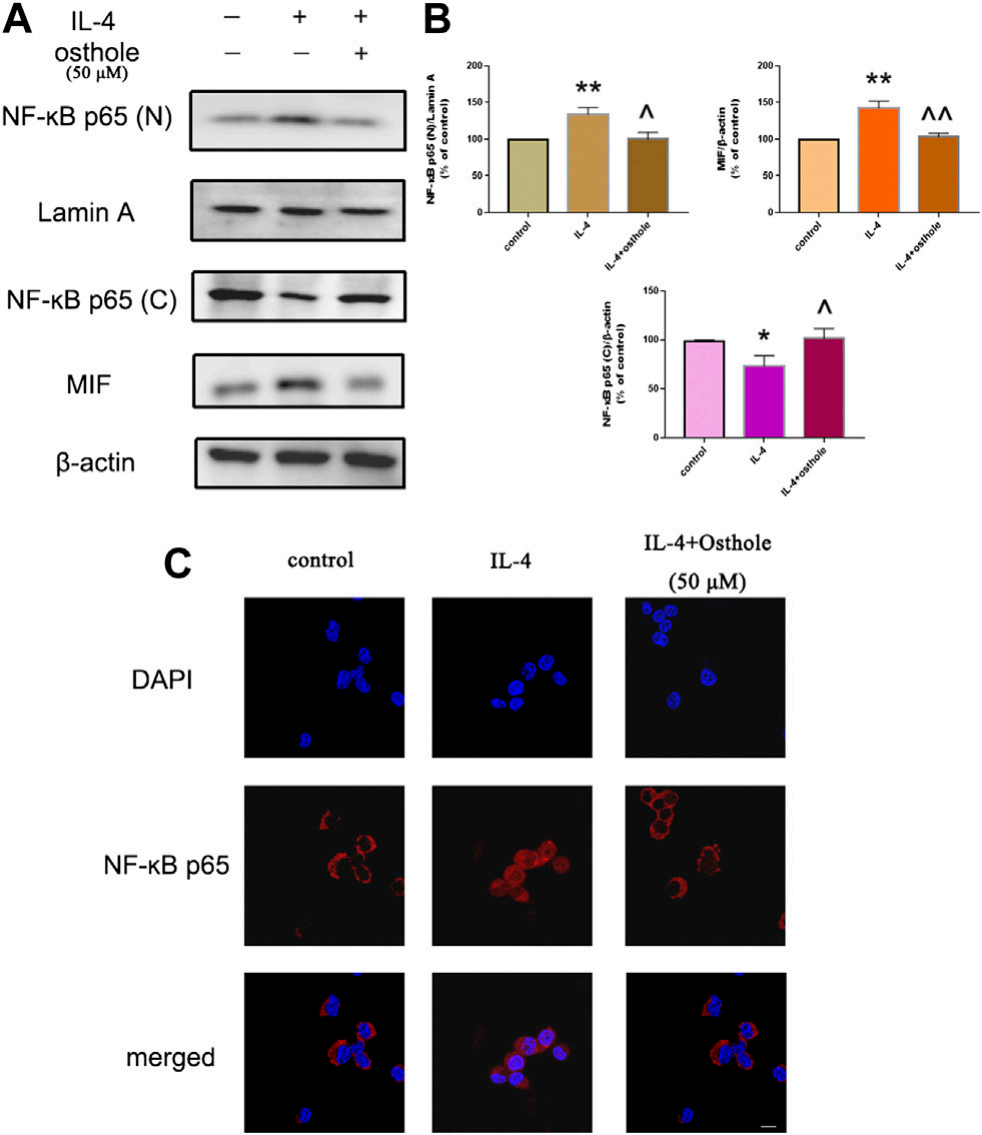
Osthole inhibits macrophage activation in an experimental model of asthma (schematic). NF-ĸB/MIF exerts a significant role in IL-4-induced macrophage activation. The biological activities of macrophage include cell proliferation, cell migration and M2-type cytokines secrete. Osthole can effectively inhibit IL-4-mediated macrophage activation.

We wished to ascertain if osthole could inhibit NF-ĸB translocation in alveolar macrophages. Cells were pretreated with osthole (50 μM) for 1 h and stimulated with IL-4 (10 ng/ml) for 24 h. Cell lysates were immunoblotted for the p65 subunit of NF-ĸB. IL-4 stimulated NF-ĸB translocation significantly, which was prevented effectively by osthole (Figures 5A,B). Immunofluorescence staining of the p65 subunit showed that osthole pretreatment prevented the IL-4-induced nuclear translocation of NF-ĸB (Figure 5C). Western blotting showed the inhibitive effect of osthole on IL-4-induced MIF expression (Figures 5A,B). These data suggested that osthole suppressed IL-4-induced NF-ĸB activation.

## Discussion

The present study elicited two novel findings: i) osthole can ameliorate airway inflammation and macrophage activation in a murine model of asthma; ii) the inhibitive effect of osthole on macrophage activation may be associated with a NF-ĸB/MIF dependent signaling pathway.

Osthole can inhibit immune inflammatory diseases such as arthritis and hepatitis (Okamoto et al., 2001; Okamoto et al., 2003). In a model of alcoholic fatty liver disease, osthole led to decreased oxidative stress and increased activation of superoxide dismutase (Zhang et al., 2011). Studies have shown that osthole attenuated lipopolysaccharide-induced acute lung injury in a murine model (Chen et al., 2013; Shi et al., 2013). Previous report indicated that the treatment with coumarins was able to cause drowsiness and stomach discomfort in asthma patients. It has been found that the maximum tolerated dose of osthole in mice was 1.50 g/kg, which showed osthole had a limited toxicity (Yang and Liu, 2012). Osthole can attenuate OVA-induced inflammation in allergic asthma by inhibiting NF-ĸB activation, but whether osthole can regulate macrophage activation in asthma (and the specific mechanism of action) is not known.

We investigated the effect of osthole on macrophage activation in OVA-induced asthma. First, we measured expression of airway inflammation-associated markers in asthmatic mice. Expression of IL-1β and TNF-α in BALF was reduced after osthole exposure. Histology also revealed inflammation and remodeling to be reduced in mice exposed to osthole. CD206 is one of the most important markers of activated M2 macrophages, so we detected the immunofluorescence of CD206 in lung tissues. We discovered that reduced expression of CD206 was involved in our OVA-challenged model of asthma after osthole treatment. We showed that osthole inhibited airway inflammation, airway remodeling and macrophage activation in our asthma model, and MIF expression was also suppressed in the osthole group. These findings suggest that osthole might be a potential anti-allergic agent for asthma treatment.

Recent studies have demonstrated that macrophages have pivotal roles in the modulation of cancer, type-II diabetes mellitus, cystic fibrosis and atherosclerosis (Sica and Mantovani, 2012; Wynn et al., 2013). Traditionally, lymphocytes, B cells, mast cells and dendritic cells are considered to be associated with a type-2 immune response, but activated M2 macrophages also contribute to the inflammation seen in asthma (Sica and Mantovani, 2012; Qian et al., 2015). Wang and colleagues demonstrated that osthole attenuated the development of pancreatic cancer in mice by inhibiting tumor-infiltrating M2 macrophages (Wang et al., 2018). We explored the role of osthole in IL-4-induced macrophage activation *in vitro*. Colony-formation and BrdU cell-proliferation assays demonstrated that osthole could suppress IL-4-induced macrophage proliferation. Scratch and Transwell assays also revealed that osthole inhibited IL-4-induced macrophage migration.

The transcriptional factor NF-ĸB has a pivotal role in the recruitment of inflammatory cells and production of Th2 cytokines in murine models of asthma (Choi et al., 2004; Kang et al., 2009). The NF-ĸB/MIF signaling pathway can regulate fatty acid-induced dysfunction and apoptosis of β-cells in pancreatic islets (Zheng et al., 2017). In the acute lung injury model, the inhibitory effect of osthole on eukocytic recruitment, and cytokine productions was regulated by NF-ĸB (Jin et al., 2018). Osthole was also proved to alleviate pulmonary arterial hypertension in monocrotaline-induced rats, which was closely associated with NF-ĸB pathway (Li et al., 2017). Osthole exerts an anti-inflammation effect in asthma by inhibiting NF-ĸB activation (Wang et al., 2017). We showed that production of IL-4-induced M2-type cytokines in macrophages was closely associated with the NF-ĸB/MIF signaling pathway. Osthole inhibited NF-ĸB activation, thereby suppressing IL-4-induced translocation of transcription factors into nuclei. These findings suggest that osthole-mediated macrophage activation in asthma may be associated with inhibition of the NF-ĸB/MIF signaling pathway, which may explain the therapeutic effects of osthole.

## Conclusion

**FIGURE 6:**
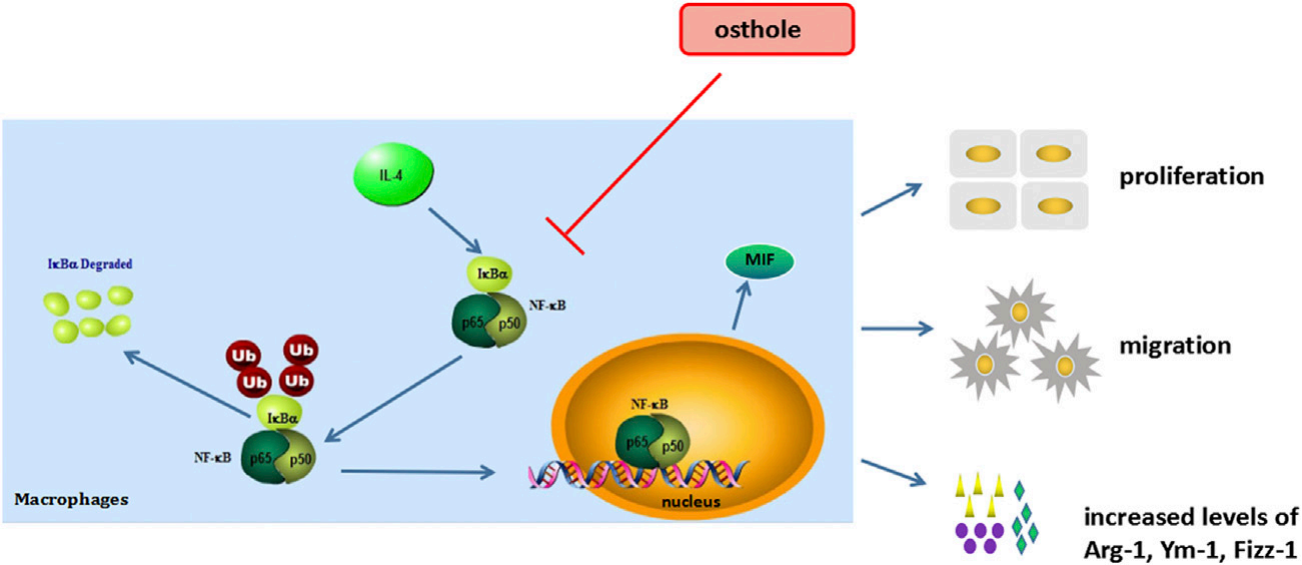
Osthole inhibits macrophage activation in an experimental model of asthma (schematic). NF-κB/MIF exerts a significant role in IL-4-induced macrophage activation. The biological activities of macrophage include cell proliferation, cell migration and M2-type cytokines secrete. Osthole can effectively inhibit IL-4-mediated macrophage activation.

We demonstrated that osthole inhibited airway inflammation and macrophage activation in a mouse model of OVA-induced asthma. Osthole could suppress IL-4-induced macrophage activation, including cell proliferation, cell migration and production of M2 cytokines. The inhibitive effects of osthole in an experimental model of asthma may be mediated by the NF-ĸB/MIF signaling pathway (Figure 6). Our findings support the potential application of osthole as a therapeutic drug against allergic asthma.

## Data Availability

The raw data supporting the conclusions of this article will be made available by the authors, without undue reservation.

## Supplementary Material

FIGURE S1 | RT-qPCR suggested that MIF expression was blocked effectively in shRNA-MIF510 transfected RAW264.7 cells. **p* < 0.05 *vs.* control.
