# Supplementary figures and images for: Osthole Attenuates Macrophage Activation in Experimental Asthma by Inhibitingthe NF-ĸB/MIF Signaling Pathway
Source: Front Pharmacol. 2021 Mar 22;12:572463. doi: 10.3389/fphar.2021.572463 (PMC8020258; doi:10.3389/fphar.2021.572463)

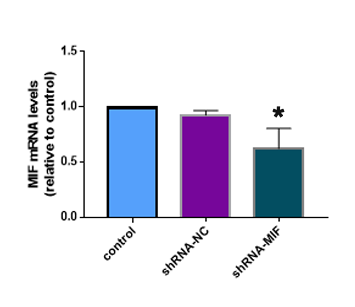

Supplement: Supplementary file 1 [file image1.tif]
